# RUNX1 and RUNX3 protect against YAP-mediated EMT, stem-ness and shorter survival outcomes in breast cancer

**DOI:** 10.18632/oncotarget.24419

**Published:** 2018-02-06

**Authors:** Madhura Kulkarni, Tuan Zea Tan, Nurfarhanah Bte Syed Sulaiman, John M. Lamar, Prashali Bansal, Jianzhou Cui, Yiting Qiao, Yoshiaki Ito

**Affiliations:** ^1^ Cancer Science Institute, NUS, Singapore; ^2^ Koch Institute for Integrative Cancer Research, Massachusetts Institute of Technology, Cambridge, MA, USA; ^3^ Yong Loo Lin Professor in Medical Oncology, NUS, Singapore; ^4^ Current address: Transnational Cancer Research Centre, Prashanti Cancer Care Mission and Indian Institute of Science Education and Research, Pune, India; ^5^ Current address: Department of Molecular and Cellular Physiology, Albany Medical College, Albany, NY, USA; ^6^ Current address: Max Planck Institute for Developmental Biology, Tübingen, Germany

**Keywords:** RUNX1 and RUNX3, YAP, breast cancer, EMT, stem-ness

## Abstract

Hippo pathway target, YAP has emerged as an important player in solid tumor progression. Here, we identify RUNX1 and RUNX3 as novel negative regulators of oncogenic function of YAP in the context of breast cancer. RUNX proteins are one of the first transcription factors identified to interact with YAP. RUNX1 or RUNX3 expression abrogates YAP-mediated pro-tumorigenic properties of mammary epithelial cell lines in an interaction dependent manner. RUNX1 and RUNX3 inhibit YAP-mediated migration and stem-ness properties of mammary epithelial cell lines by co-regulating YAP-mediated gene expression. Analysis of whole genome expression profiles of breast cancer samples revealed significant co-relation between YAP–RUNX1/RUNX3 expression levels and survival outcomes of breast cancer patients. High RUNX1/RUNX3 expression proved protective towards YAP-dependent patient survival outcomes. High YAP in breast cancer patients’ expression profiles co-related with EMT and stem-ness gene signature enrichment. High RUNX1/RUNX3 expression along with high YAP reflected lower enrichment of EMT and stem-ness signatures. This antagonistic activity of RUNX1 and RUNX3 towards oncogenic function of YAP identified in mammary epithelial cells as well as in breast cancer expression profiles gives a novel mechanistic insight into oncogene–tumor suppressor interplay in the context of breast cancer progression. The novel interplay between YAP, RUNX1 and RUNX3 and its significance in breast cancer progression can serve as a prognostic tool to predict cancer recurrence.

## INTRODUCTION

Yes-associated protein (YAP), is a transcriptional co-activator that functions downstream of Hippo-tumor suppressor pathway. Deactivation of the Hippo pathway leads to nuclear translocation of YAP. Increased nuclear YAP has been associated with increased cancer risk and poor patient survival in many solid tumors like liver cancer, lung cancer, head and neck cancer and colon cancer; reviewed in [1, 2]. Cell-based studies and mouse xenograft studies have correlated YAP as a potential oncogene in breast cancer [3–5] as well an effector of metastasis [6, 7].

YAP, as a transcriptional co-activator binds to specific DNA-binding proteins in a context dependent manner. For example, YAP-TEAD interaction has been demonstrated to be essential for the oncogenic activity of YAP [5, 8]. While, YAP and p73 transcriptional complex is known to drive apoptosis in response to DNA-damage [9, 10]. The first demonstration of YAP's transcriptional co-activator function was made in the context of RUNX transcriptional reporter, with the direct interaction between YAP and RUNX transcription factors [11]. However, unlike YAP-p73 or YAP-TEAD, functional consequences of YAP-RUNX interaction have not been explored in detail.

RUNX transcription factors are a family of proteins with a highly conserved DNA binding ‘Runt’ domain and they play important role in diverse biological processes; reviewed in [12]. Aberrant expression of RUNX proteins and mutations in RUNX genes have been extensively linked with carcinogenesis in gastric, lung, colon, skin and blood as reviewed by Chuang *et al*. [13]. Out of the three RUNX proteins, RUNX1 and RUNX3 have been recently identified as novel tumor suppressors in breast cancer. Recurrent mutations in RUNX1 and its binding partner CBFβ were detected in two independent cohorts of breast cancer deep sequencing studies [14, 15]. RUNX3 is frequently inactivated by hypermethylation in human breast cancers and its haploinsufficiency has been linked with spontaneous breast carcinoma in mice [16, 17]. Despite this emergent role of RUNX1 and RUNX3 as tumor suppressors in breast cancer, the mechanistic details regarding how they exert their tumor suppressor function in breast cancer is yet to be understood.

Given the recent discovery of RUNX1 and RUNX3 as tumor suppressors in the context of breast cancer and their known interaction with oncogene-YAP, we investigated whether YAP-RUNX interaction plays any role in molecular pathogenesis of breast cancer. Effect of RUNX1 or RUNX3 expression on YAP-mediated oncogenic transformation of mammary epithelial cells was assessed followed by gene expression analysis. Using the gene enrichment set derived from the mammary epithelial cell model, whole genome expression profiles of 3992 breast cancer patients; compiled from global datasets, were analyzed for co-relation of YAP and RUNX1-RUNX3 expression towards survival and metastatic parameters.

## RESULTS

### RUNX3 attenuates YAP-induced proliferation and migration in mammary epithelial cells

RUNX transcription factors are known to directly interact with YAP and co-regulate transcription, where YAP acts as a co-activator for RUNX transcriptional reporter [11]. RUNX proteins interact via their PY motif with WW domain of YAP [11, 18]. To investigate physiological relevance of YAP-RUNX interaction in the context of breast cancer progression, we tested effect of co-expression of RUNX3 on YAP-mediated pro-tumorigenic transformation of mammary epithelial cells. The non-transformed mammary epithelial cell line; MCF10a was transduced with retrovirus(es) for stable expression of YAP, RUNX3 and co-expression of YAP and RUNX3.

**Figure 1:**
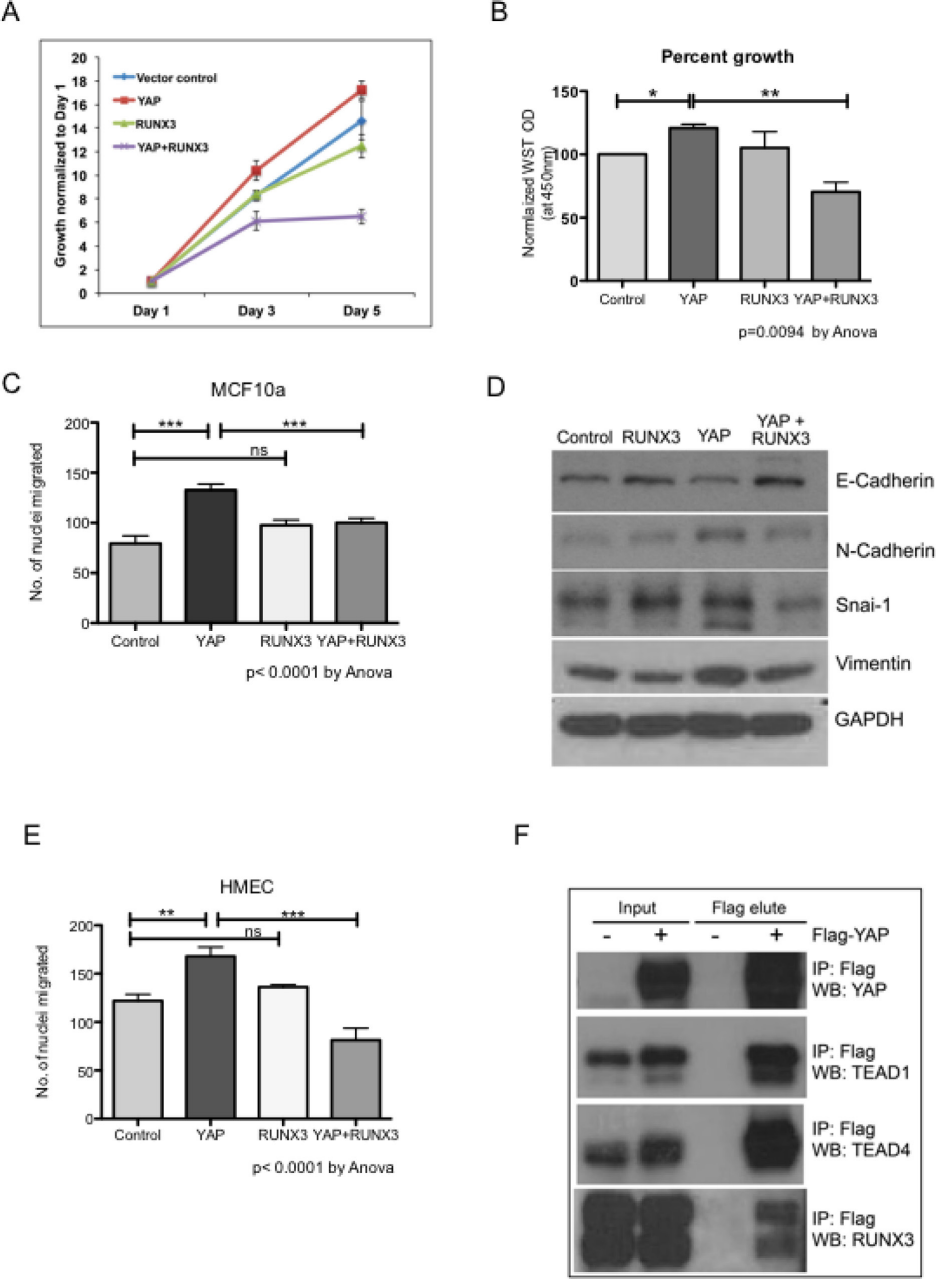
RUNX3 attenuates YAP-induced proliferation and migration RUNX3 co-expression suppresses YAP-induced proliferation and migration in mammary epithelial cells. (**A**) Cell viability was measured from triplicate wells using WST reagent for stable MCF10a cells on day 1, 3 and 5. WST readings normalized to their respective day 1 readings are plotted in the growth curve. (**B**) Average WST readings, normalized against vector control from three independent experiments are plotted. Error bars represent ± SEM. For *P*-value, one-way Anova test was performed using graph pad prism followed by Newman–Keuls multiple comparison test. (**C**) Transwell migration assay MCF10a stable cell lines. Average number of nuclei migrated through 8-micron membrane within 24 hours are plotted for each stable cell line. The assay was conducted with triplicate trans-wells per stable cell line in three independent experiments. The error bars represent ± SEM. Statistical significance is calculated using one-way Anova followed by Newman–Keuls multiple comparison test. (**D**) Western analysis for representative epithelial (E-cadherin) and mesenchymal (N-cadherin, Vimentin and Snai1) markers from the whole cell lysates of stable MCF10a cell lines. GAPDH expression levels indicate equal loading. (**E**) Transwell migration assay HMEC stable cell lines. The experimental details are identical to Figure 1C. Average number of nuclei migrated from three independent experiments are plotted, and one way Anova followed by Newman–Keuls multiple comparison test was performed for statistical significance. (**F**) Co-immunoprecipitation using Flag beads was performed for 1 mg of 293T whole cell lysates with or without Flag-YAP transfection. Immnoprecipitated proteins were eluted by flag peptide competition. 30 μg of input proteins and 50% of flag peptide elute were run on SDS-PAGE. Endogenous TEAD1, TEAD4 and RUNX3 co-immunoprecipitated with Flag-YAP are detected on a western blot.

YAP is known to induce proliferation and epithelial mesenchymal transition (EMT) in mammary epithelial cells [3, 5]. Stable expression of YAP in MCF10a lead to increased proliferation as previously reported (Figure 1A and 1B). Co-expression of RUNX3 with YAP significantly inhibited YAP-mediated proliferation of MCF10a (Figure 1A and 1B). Expression of YAP and RUNX3 in stable MCF10a assessed by western blot is shown in Supplementary Figure 1. YAP induced trans-well migration in MCF10a (Figure 1C) with concomitant increase in protein expression of mesenchymal markers like N-cadherin, Vimentin and Snai-1 (Figure 1D). RUNX3 co-expression with YAP inhibited YAP-induced migration and mesenchymal marker expression in MCF10a (Figure 1C–1D). A similar inhibition of YAP-mediated trans-well migration by RUNX3 was observed in HMEC cells (Figure 1E). We performed co-immunoprecipiation assay to confirm the interaction between YAP and RUNX3. Flag-YAP was immunoprecipitated with Flag-beads and co-immunoprecipitation of TEAD1 and TEAD4 (Figure 1F) ensured successful immunoprecipitation of YAP. Co-immunoprecipiation of RUNX3 with Flag-YAP confirmed the interaction between the two proteins (Figure 1F), indicating that RUNX3 mediated inhibition could possibly be dependent on YAP-RUNX3 interaction.

### RUNX3 as well as RUNX1, suppress YAP-induced Mammosphere formation in an interaction dependent manner

**Figure 2:**
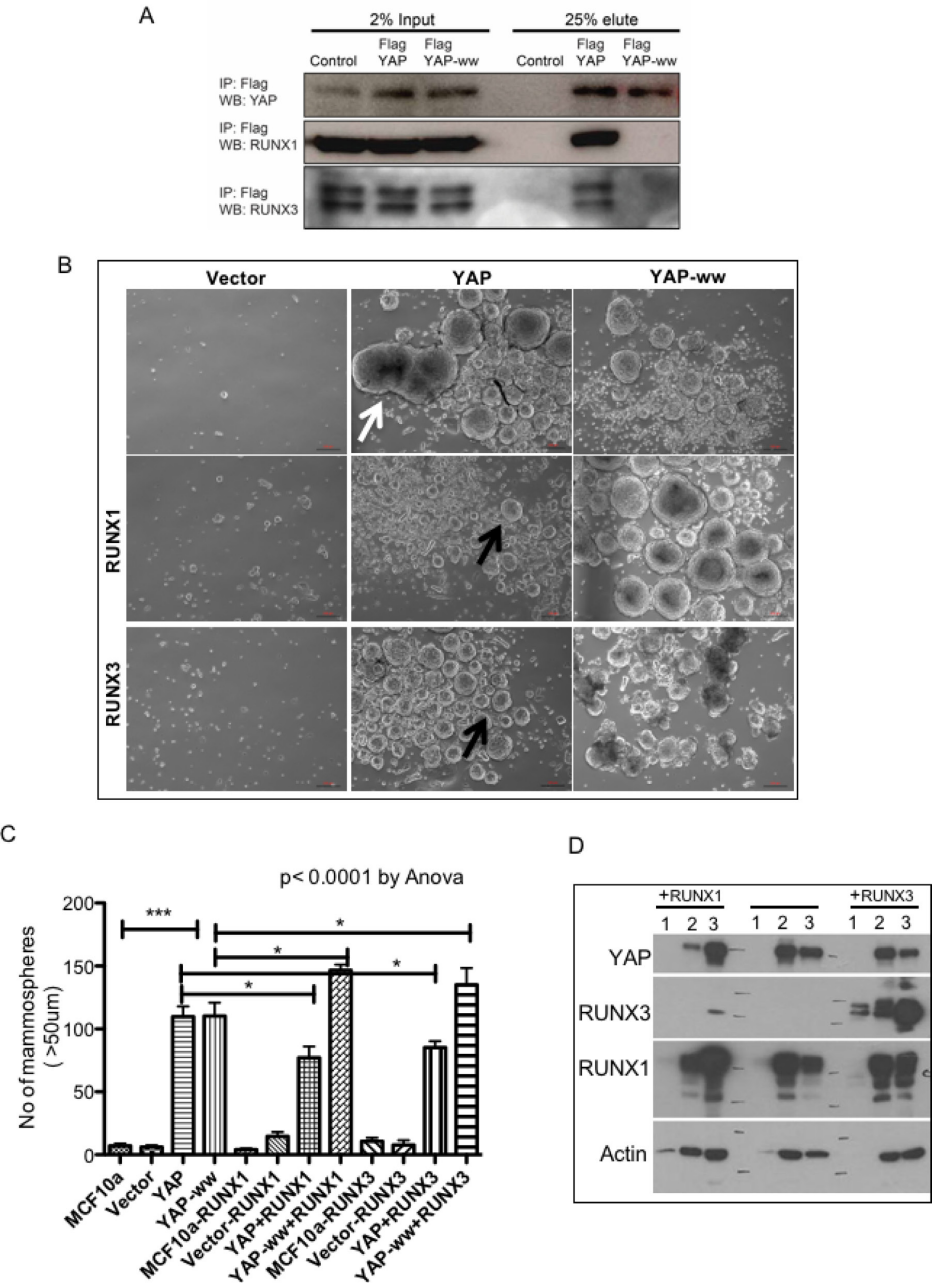
RUNX1 and RUNX3 suppress YAP-induced Mammosphere formation in an interaction dependent manner (**A**) 293T cells were co-transfected with RUNX3-mCherry or RUNX1-GFP with either control vector or Flag-YAP or Flag-YAP-ww. Whole cell extracts were subjected to immunoprecipitation and co-immunopreciptated proteins were eluted by Flag peptide competition. 25 μg of input proteins and 25% of eluted proteins were run on SDS-PAGE. Co-immunoprecipitated YAP and RUNX proteins are detected by western analysis using YAP and RUNX specific antibodies, respectively. (**B**) Representative images of Mammosphere cultures of stable MCF10a expressing control vector (MSCV) or YAP or YAP-ww (top row) and co-expressing RUNX1 (middle row) or RUNX3 (bottom row). Stable expression of YAP leads to formation of enlarged Mammosphere as indicated with white arrow. Co-expression of RUNX proteins suppresses YAP induced Mammosphere size enlargement (black arrows). Co-expression of RUNX with YAP-ww does not show suppression of Mammosphere size. DIC images were taken at 4X magnification on day 11. Scale bar represents 100 μm. (**C**) Average number of Mammosphere (>50 μm) from duplicate wells run in two independent experiments are plotted for each stable MCF10a cell line. The error bars represent standard error of mean. One-way Anova followed by Newman–Keuls multiple comparison test calculated statistical significance. (**D**) Western analysis of 20 μg whole cell extracts from Mammosphere harvested on day 11. The lane numbers represent, 1: MSCV (empty vector), 2: YAP, 3: YAP-ww. Left panel; ‘+RUNX1’ cells co-express RUNX1 and right panel; ‘+RUNX3’ cells co-express RUNX3 along with 1–3. Vector control MCF10a do not form mammosphere, hence total protein extracted is too low to detect protein expression.

YAP and RUNX3 are known to interact via their WW domains and PY motif, respectively [11, 18]. To test whether RUNX3 mediated inhibition of YAP-induced phenotypes is dependent on YAP-RUNX3 interaction, we employed WW mutant YAP: W^199^F and W^258^F [19] to abolish its interaction with RUNX3. YAP or WW mutant YAP-ww was co-expressed with RUNX3 or RUNX1 in 293T cells and their interaction was assessed by co-immunoprecipitation. Western analysis confirmed that YAP indeed co-immunoprecipitated with RUNX3 and RUNX1, but not YAP-ww (Figure 2A), suggesting intact WW domains is required for the interaction of YAP with RUNX3 as well as RUNX1.

To test whether suppression of YAP-induced pro-tumorigenic phenotypes by RUNX3 is dependent on direct interaction between YAP and RUNX3, RUNX3 was co-expressed with YAP or YAP-ww in MCF10a. We included RUNX1 co-expressing stable MCF10a as well to investigate if both tumor suppressor proteins; RUNX3 and RUNX1 abrogate YAP-mediated oncogenic phenotypes in MCF10a. We chose RUNX1 and not RUNX2, since RUNX1 together with its binding partner CBFb has recently been discovered to be mutated ~ 4–6% in breast cancers; suggesting its tumor suppressor function in this context [14, 15].

Effect of RUNX3 or RUNX1 co-expression on YAP-mediated induction of mammosphere formation was tested as a read out for stem-ness. MCF10a cells do not form mammosphere [20]. Stable expression of YAP or YAP-ww in MCF10a induced large mammosphere (>50-micron) within 11 days (Figure 2B–2C) suggesting increased stem-ness properties. RUNX3 or RUNX1, both significantly inhibited the number of mammosphere formed when co-expressed with YAP but not with YAP-ww (Figure 2B–2C). This data suggests that RUNX-YAP interaction via WW domain of YAP is necessary for suppression of YAP-induced mammosphere formation. Mammosphere were harvested at the end of the assay (day 11) to confirm stable expression of YAP and RUNX proteins (Figure 2D).

### RUNX3 and RUNX1 suppress YAP-induced aberrations in mammary acini in an interaction dependent manner

*In vitro* differentiation of mammary lobules from normal epithelial cells like MCF10a on reconstituted basement membrane (matrigel) has been used as a model to study possible aberrations leading to breast tumor initiation [21, 22]. Single cells seeded onto matrigel proliferate and differentiate into polarized spheres with epithelial cells encompassing hollow lumen; referred as acini [23]. Oncogenes like ERBB2, Myc, K-Ras have been shown to interfere with normal acini differentiation either by enhancing proliferation, by altering polarity of the epithelial cells or by suppressing apoptotic signaling as reviewed [24].

**Figure 3:**
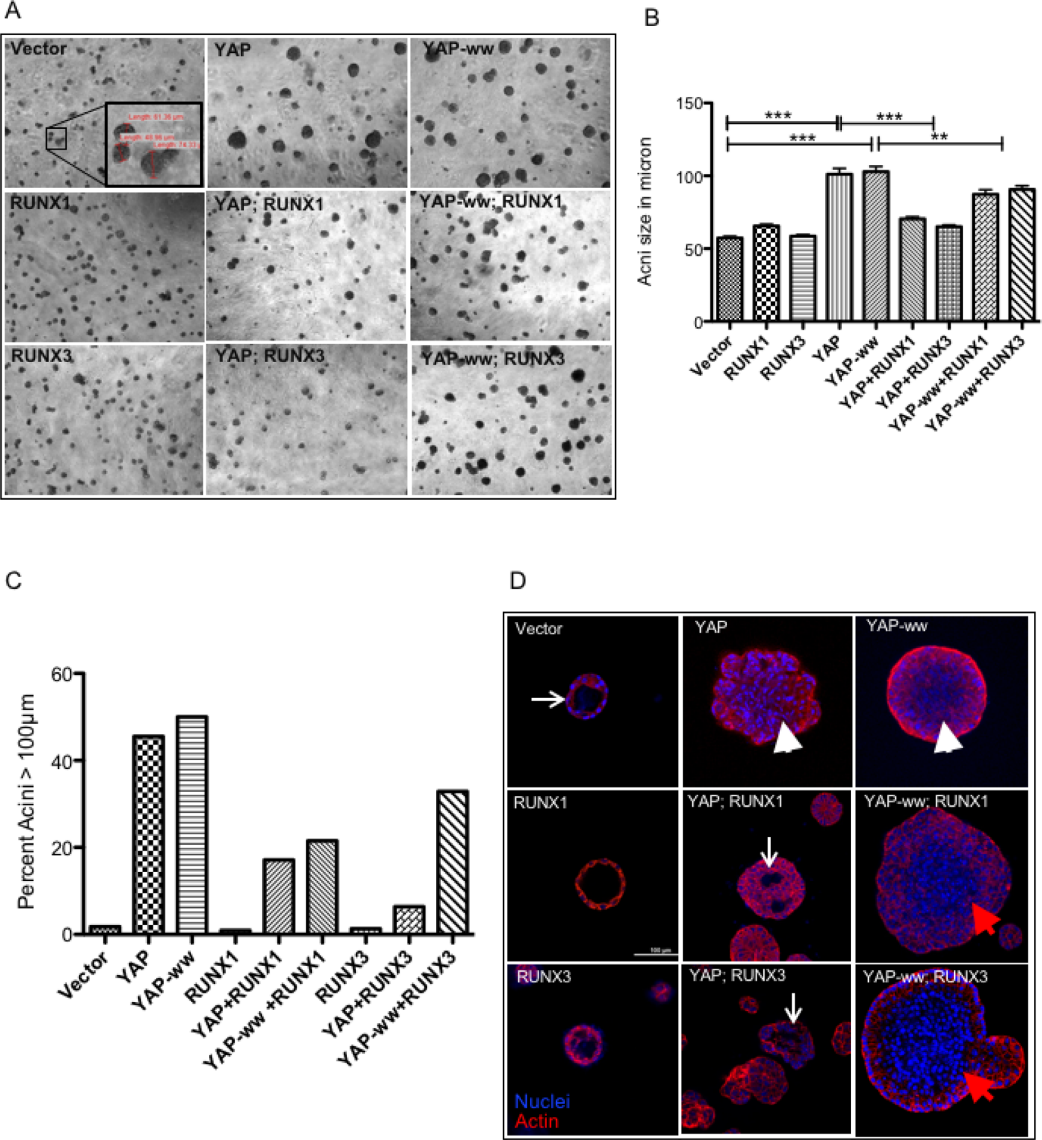
RUNX1 and RUNX3 suppress YAP-induced aberrations in mammary acini in an interaction dependent manner (**A**) Representative images of mammary acini structures, grown on matrigel from single cell cultures of stable MCF10A cell lines. DIC images were taken on day of the cultures under 4× magnification. The inset image in the vector control panel shows representative acini with their corresponding diameter in μm, measured using Olympus Cell^F^ software. (**B**) Diameter of 100–150 acinar structures was measured on day 9 and the average values are plotted for each stable cell line. The error bars represent standard error of mean and the *p* values of statistical significance denoted above, are calculated using one-way Anova followed by Newman–Keuls multiple comparison test. (**C**) The percentage of acini larger than 100 μm in size are plotted for each stable cell line. Majority of acini formed by vector control or RUNX1 or RUNX3 stable cell lines are smaller (<100 μm) in size. YAP or YAP-ww stable expression leads to more than 50% of acini to grow larger than 100 μm in size. Co-expression of RUNX1 or RUNX3 suppresses YAP induced size enlargement. (**D**) Confocal images of individual acinus immunostained for actin (phalloidin, red) and DNA (DAPI, blue), taken at 40× magnification. White arrows point to the lumen formation in vector control, YAP+RUNX1 and YAP+RUNX3 panel. The lack of lumen formation in the acinus is indicated with white arrowheads, panel YAP, YAP-ww+RUNX1 and YAP-ww+RUNX3 acini. Scale bar represents 100 μm.

In our assays, expression of YAP altered normal acini development. Acini derived from the vector control cells ceased growth at average ~50 μm size, while acini expressing either YAP or YAP-ww continued their growth beyond 50 μm (Figure 3A). On day 9, acini formed by the vector control cells were 56 μm (SD ±13) in size, while acini expressing either YAP or YAP-ww were of 103 μm (±41) and 102 μm (±35) size, respectively (Figure 3B). The increase in size indicates that stable expression of YAP is sufficient to induce morphogenic aberrations during acini differentiation. Co-expression of either RUNX1 or RUNX3 with YAP reduced the average size of the acini to 75 μm (±25) and 65 μm (±20), respectively (Figure 3B). In contrast, RUNX1 or RUNX3 co-expression with YAP-ww did not result in such a sharp reduction of size (average size 84 μm and 91 μm respectively) (Figure 3A–3C). About 50% of the acini expressing YAP or YAP-ww were within the 100–200 μm range. While only 17% or 6% of the acini reached more than 100 μm size when co-expressed with YAP and RUNX1 or RUNX3, respectively (Figure 3C).

Confocal imaging of the acini on day 16 revealed that the acini derived from YAP or YAP-ww expressing cells had no lumen formation (Figure 3D, arrowheads) compared to that of vector control (Figure 3D, arrow). Interestingly, RUNX1 or RUNX3 co-expression with YAP but not with YAP-ww, partially induced lumen formation (Figure 3D).

### RUNX1 and RUNX3 alter YAP target gene expression in an interaction dependent manner

**Figure 4:**
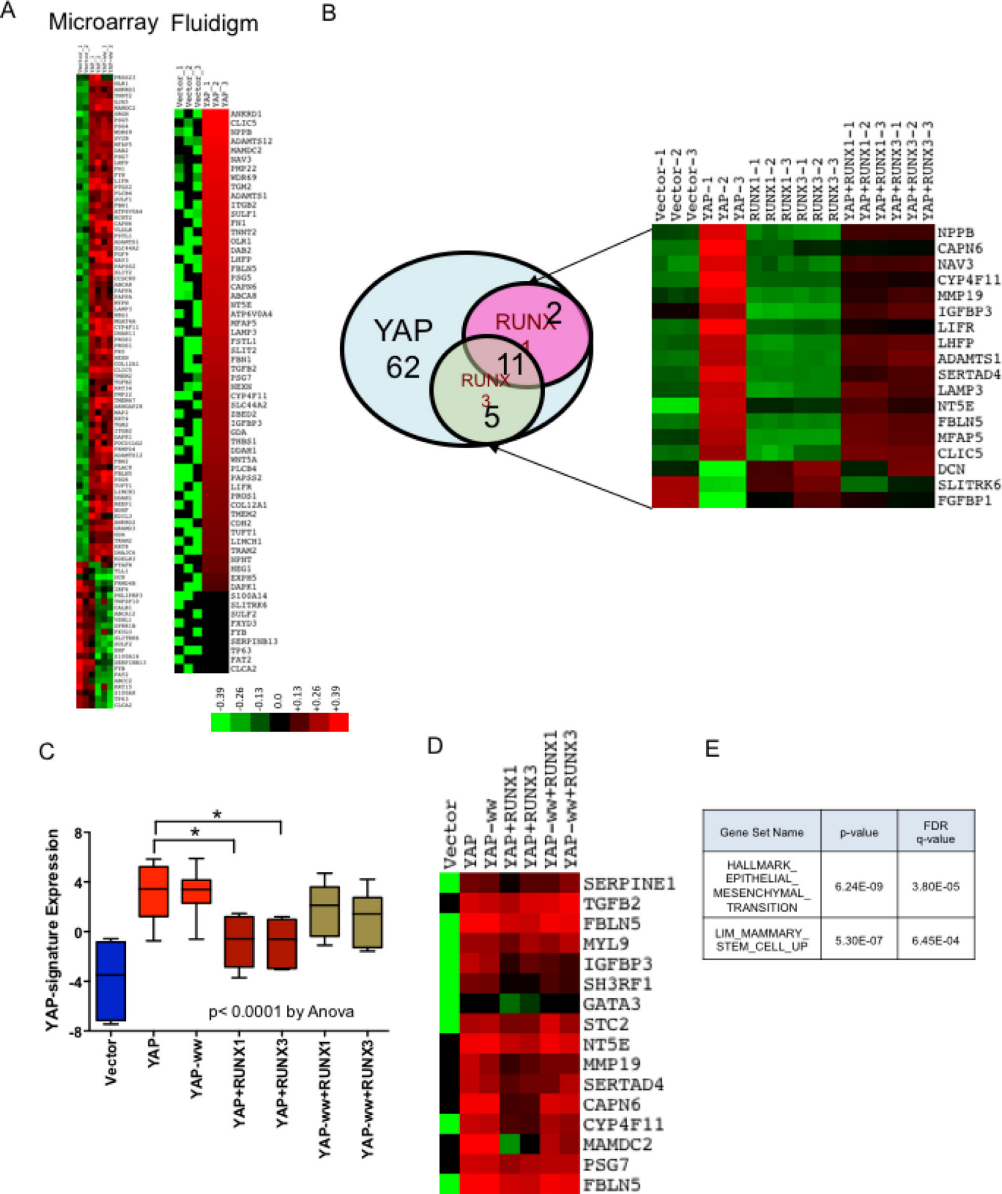
RUNX1 and RUNX3 alter YAP target gene expression Microarray gene expression analysis was performed for MCF10a stable cell lines expressing YAP or YAP-ww with or without RUNX1/RUNX3. Followed by Fluidigm RT-PCR, to confirm differential gene-expression. RNA from each cell line was run in triplicate for both, microarray and Fluidigm RT-PCR. (**A**) Differential expression of 104 genes that are altered by +/− 2 fold in YAP or YAP-ww expressing MCF10a, analyzed by microarray (on left). Differential expression of 62 out of 104 genes, confirmed by Fluidigm RT-PCR to be +/−2 fold in YAP or YAP-ww stable cell lines (on right), referred as YAP-signature. Red denotes up-regulation while green denotes down-regulation of gene expression. Color key refers to log expression values. (**B**) RUNX1 or RUNX3 co-expression with YAP negatively regulates a subset of YAP transcriptional targets, shown in the Venn diagram. Out of 62 genes in the YAP-Signature (blue circle), 29% (18) of the genes are negatively regulated by RUNX1 (pink circle) and/or RUNX3 (green circle). Differential expression of genes co-regulated by YAP and RUNX1/RUNX3 in each stable cell line is shown in the heat-map. (**C**) Boxplot of gene expression in ΔΔCTs of RUNX1 and RUNX3 co-regulated YAP transcriptional targets. Whiskers indicate min and max values, whereas box indicates 1st quartile, median and 3rd quartile. The error bars represent standard error of mean. Statistical significance is calculated using one-way Anova, with *p* value < 0.0001. Statistical significance of YAP signature expression in presence and absence of RUNX1 or RUNX3 is assessed by unpaired *t*-test. *p* values are denoted above the bar graphs for respective comparisons. (**D**) Heat-map expression of YAP-signature genes that are co-regulated by RUNX1 or RUNX3 when co-expressed with YAP but to a lesser degree with YAP-ww. Each column of heat-map represents average expression from triplicates. (**E**) YAP-Signature genes that were altered by RUNX1 and/or RUNX3 were analyzed for the enrichment of molecular signatures using MSigDB version 5.2. Assessment for overlap with signatures revealed the top two significant gene signatures to be EMT and mammary stem-cell signatures, as shown in the table.

Both, YAP and RUNX are transcriptional regulators and Yagi *et al.* (1999) had demonstrated that YAP and RUNX co-regulate expression of RUNX reporter. Hence, we speculated that co-expression of RUNX1 or RUNX3 could alter the transcriptional profile of YAP target genes. To validate, microarray analysis was performed with the total RNA isolated from stable MCF10a cell lines using Affymetrix Human Gene 1.0 ST array (See methods). After RMA normalization, overexpression of YAP, RUNX3 and RUNX1 was confirmed in respective stable cell lines (Supplementary Figure 2). 104 genes were identified that are ±2 fold enriched in YAP and YAP-ww expressing stable MCF10a cell lines, compared to that of vector control (Figure 4A). To validate, high-throughput RT-PCR was carried out using BioMark 48 × 48 gene expression platform (Fluidigm) for the 104 target genes. Amongst these, 62 genes were confirmed to be up or down regulated by 2-fold in both YAP and YAP-ww expressing stable cell lines (Figure 4A). These 62 genes are designated as ‘YAP-signature’ genes (Supplementary Table 1) and the statistical significance of the change in expression is provided in Supplementary Table 2. The ‘YAP-signature’ gene expression was then projected on the rest of the stable cell lines’ gene expression profiles. Co-expression of RUNX1 or RUNX3 with YAP indeed altered expression of 16 and 13 genes; respectively, with 11 genes commonly altered by both (Figure 4B). This repression of YAP-signature genes by RUNX1 or RUNX3 is observed with YAP, but not with YAP-ww (Figure 4C). Indicating, direct interaction between RUNX proteins and YAP is required for RUNX mediated alteration of YAP-signature gene expression. The loss of repression by RUNX1 or RUNX3 in the context of YAP-ww mutant is reflected in the heat map of selective YAP-signature genes (Figure 4D). Thus, co-regulation of YAP target gene expression by RUNX1 or RUNX3 is dependent on direct interaction between YAP and RUNX proteins. YAP-Signature genes that were altered by RUNX1 and/or RUNX3 were analysed for the enrichment of molecular signatures using MSigDB. Assessment for overlap with hallmark signatures revealed the top two significantly represented gene signatures to be EMT (M5930) and mammary stem-cell (M2573) [25] signatures (Figure 4E).

### RUNX3 expression attenuates migration and mammosphere formation in breast cancer cell line only when YAP expression is high

**Figure 5:**
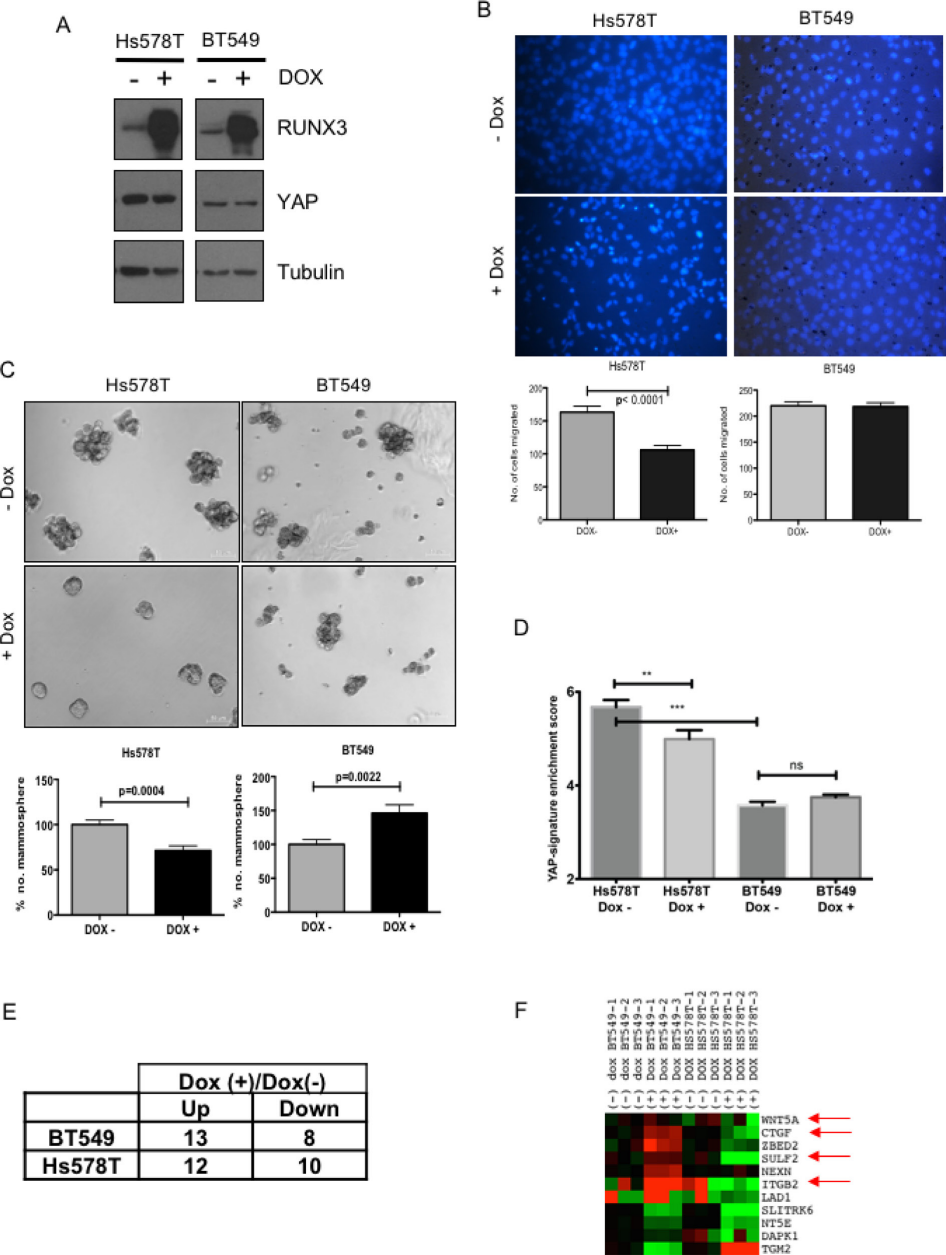
RUNX3 expression attenuates migration and mammopshere formation in breast cancer cell line with high-YAP expression (**A**) Western analysis of Hs578T and BT549 breast cancer cell lines; infected with doxycycline inducible RUNX3. Sub-confluent cells cultured with +/− 1 μg/ml doxycycline for 24 hrs were harvested and analyzed for RUNX3, YAP and tubulin expression on SDS-PAGE. (**B**) Trans-well migration assay for Hs578T and BT549 cell lines with or without doxycycline inducible RUNX3 expression. Average number of nuclei migrated overnight from three independent experiments are plotted. Error bars represent standard error of mean. Statistical significance is calculated using unpaired student's *t*-test. (**C**) Average number of Mammosphere (>50 μm) counted on day 11, from triplicate experiments are plotted for Hs578T and BT549 stable cell lines cultured with +/− 1 μg/ml doxycycline. The numbers are normalized to mean mammosphere formed without doxycycline (−Dox). The error bars represent standard error of mean. Statistical significance is calculated using unpaired student's *t*-test. (**D**) Fluidigm RT-PCR was performed for BT549 and Hs578T cell lines with and without doxycycline induction for 62 YAP-signature genes. Enrichment of YAP-signature gene expression for both the cell lines with and without doxycycline induction is plotted. One-way Anova was performed for statistical significance, significance values for pair-wise samples determined by Newman–Keuls multiple comparison test are reflected on the graph. (**E**) The number of genes up or down regulated by 2-fold after doxycycline induction in Hs578T and BT549. (**F**) Differential expressions of the genes that are commonly altered after RUNX3 expression in both the cell line pairs are plotted in the heat-map. The arrows indicate the genes that are oppositely regulated by RUNX3 overexpression in BT549 vs Hs578T and are known to have implications in breast cancer progression.

Further, we investigated whether expression of RUNX can abrogate EMT and stem-ness in breast cancer cell lines and whether this abrogation is dependent on YAP expression. RUNX3 has been shown to inhibit MDA-MB-231 invasion and growth of xenograph tumors in nude mice [26]. Hence, we chose breast cancer cell lines other than MDA-MB-231 to confirm whether RUNX3 mediated inhibition of tumorigenic phenotypes depends on the level of YAP expression. Hs578T and BT549 (Figure 5A) were chosen for the study, as the extent of YAP expression differed in the two cell lines. Hs578T reflected higher levels of nuclear YAP expression, compared to that of BT549 (Figure 5A). The two cell lines were transduced with retroviral construct expressing doxycycline-inducible RUNX3. Induction of RUNX3 expression upon doxycycline treatment was confirmed by western analysis (Figure 5A).

To assess for the effect of RUNX3 on EMT, migratory properties of the cell lines were tested using transwell migration with or without doxycycline induction. RUNX3 expression significantly down-regulated trans-well migration of high-YAP expressing Hs578T, but not that of low-YAP expressing BT549 (Figure 5B). For assessing effect on stem-ness properties, mammosphere formation assay was performed for both the cell lines with or without doxycycline induction. Similar to migration, mammosphere forming capacity of Hs578T was also compromised significantly after doxycycline induced RUNX3 expression (Figure 5C). Whereas low-YAP expressing BT549 cell line showed marked increase in mammosphere forming capacity with RUNX3 induction (Figure 5C).

To confirm whether RUNX3 expression altered YAP target gene expression in Hs578T and BT549, high through-put RT-PCR was performed for both the cell lines with and without doxycycline induction using BioMark platform (Fluidigm) for YAP-Signature gene set. As expected, Hs578T reflected significantly higher enrichment of YAP-signature genes compared to BT549 and doxycycline induced RUNX3 down-regulated the enrichment of YAP-signature genes in Hs578T cell line significantly, but not in BT549 (Figure 5D). Out of 62 YAP-signature genes tested, total of 22 and 21 genes were altered (± 2-fold) after RUNX3 overexpression in Hs578T and BT549; respectively (Figure 5E). Comparative analysis revealed that 11 of these genes were commonly regulated in both the cell lines. Interestingly, 7 out of these 11 genes were repressed by RUNX3 overexpression in Hs578T, high-YAP expressing cells (Figure 5F). Whereas in low-YAP expressing BT549, these 7 genes were either up-regulated or did not alter in expression after RUNX3 overexpression (Figure 5F). The list of the genes and the statistical significance of the change in expression after RUNX3 overexpression is provided in Supplementary Table 3. The 7 genes include CTGF, ZEBD2, SULF2 and integrin B2, Wnt5A. Down regulation of these genes in Hs578T co-relates well with RUNX3 mediated inhibition of migration and stem-ness. While, up-regulation of CTGF and ZEBD2 after RUNX3 overexpression could explain increased mammosphere formation in BT549.

We also knocked down YAP expression in Hs578T and BT549 cell lines (Supplementary Figure 3A) to confirm that phenotypic effects of RUNX3 overexpression are indeed resulting from abrogation of YAP function. After YAP-knock-down, YAP expression was down-regulated in both the cell lines (Supplementary Figure 3A). Mammosphere formation was affected only in Hs578T and not in BT549, in fact mammosphere formation by BT549 was significantly enhanced after YAP knock-down (Supplementary Figure 3B). Similar effect of RUNX3 overexpression on mammosphere formation by Hs578T and BT549 is observed (Figure 5C) indicating, effect of RUNX3 is most likely mediated by modulating YAP function.

These data reveal that tumor suppressive effect of RUNX3 expression on breast cancer cell lines is manifested specifically in the context of elevated YAP expression. Pro-tumorigenic properties like EMT and stem-ness, which are directly linked with tumor metastasis and progression, are suppressed by RUNX3 in YAP expression dependent manner, similar to what we observed in MCF10a cell line.

### YAP-signature expression levels stratify breast cancer patients’ survival outcomes together with RUNX1-RUNX3 expression

To assess for the clinical significance of RUNX3 and RUNX1 mediated suppression of YAP-mediated oncogenic function, we analyzed available expression dataset of breast cancer patients. Publicly available breast cancer expression cohorts annotated with survival and disease free outcomes were downloaded from Gene Expression Omnibus (GEO) and ArrayExpress. The list of cohorts and normalization method can be found in methods section. The standardized data yielded a dataset of 3992 breast cancer tumors, and 22 normal breast tissue samples.

**Figure 6:**
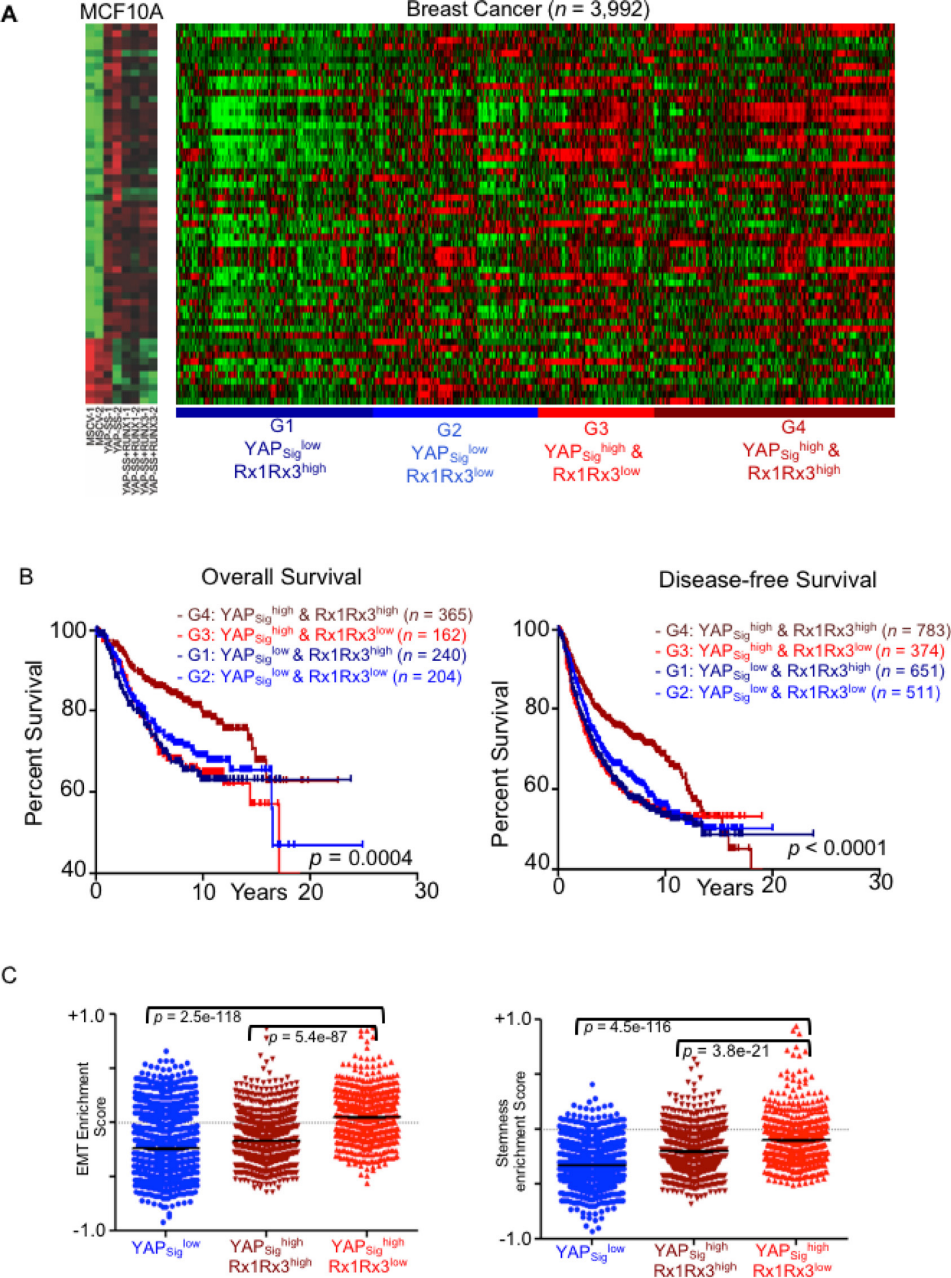
High YAP-signature predicts survival outcomes of breast cancer patients in RUNX expression dependent manner YAP-signature derived from MCF10A stable cell lines expressing constitutively nuclear YAP is projected on the breast cancer expression dataset of 3992 patient samples. (**A**) Unsupervised hierarchical clustering was performed using Cluster 3.0 software. Heatmap on the left shows gene expression levels of YAP-signature in vector control, high-YAP (YAP) and high-YAP; high-RUNX (YAP+RUNX1 and YAP+RUNX3) MCF10A. The heatmap on the right shows individual breast cancer patient's expression panel clustered according to YAP-signature. The clusters G1-G4 are named based on YAP signature enrichment and RUNX1/RUNX3 expression levels. (**B**) Kaplan–Meier analysis for overall (left) and disease-free (right) survival outcomes of the patient clusters according to YAP-signature enrichment and RUNX1-RUNX3 expression levels. Statistical significances are computed using log-rank test indicated at the bottom. (**C**) ssGSEA analysis performed for the computation of enrichment score (*y*-axis; mean ± SEM) of EMT (left) and stem-ness (right) signatures for individual the breast cancer gene expression dataset. Enrichment scores for individual patient are plotted (each dot) according to their YAP-signature cluster association. Statistical significance is calculated using the Mann–Whitney test.

We reasoned that enrichment score for ‘YAP-signature’ genes within each individual tumor would better indicate the extent of functional role of YAP. Hence, YAP-signature gene expression as a reflection of oncogenic activity of YAP was analyzed for association with clinical parameters. Unsupervised hierarchical clustering of the breast cancer patient expression data (3992 patient samples) was performed based on the similarity with the ‘YAP-signature’ (Figure 6A). Unsupervised hierarchical clustering identified four subgroups, G1-G4. Two subgroups (G3 and G4) with higher enrichment of YAP-signature are referred as YAP_sig_^high^, while the two with no significant enrichment (G1 and G2) are referred as YAP_sig_^low^ (Supplementary Figure 4A and Figure 6A).

In our cell-based assays, expression of RUNX1 or RUNX3 significantly inhibited YAP mediated pro-oncogenic phenotypes. Hence, we investigated whether RUNX1-RUNX3 expression levels influence survival outcomes of breast cancer patients within YAP-signature cohorts. Average RUNX1-RUNX3 expression in four YAP-signature based cohorts was noted (Supplementary Figure 4B). The YAP_sig_^high^ and YAP_sig_^low^ subgroups were further distinguished based on their RUNX1-RUNX3 expression as G4: YAP_sig_^high^Rx1Rx3^high^, G3: YAP_sig_^high^ Rx1Rx3^low^, G1: YAP_sig_^low^ Rx1Rx3^high^, and G2: YAP_sig_^low^ Rx1Rx3^low^.

Kaplan–Meier analysis was performed for these four cohorts to assess overall and disease-free survival (DFS) defined as progression free and local or distant metastasis free survival. Higher RUNX1-RUNX3 expression within YAP-signature-high cohort indeed reflected protective effect towards overall and disease-free survival. G4: YAP_sig_^high^ Rx1Rx3^high^ cohort showed significantly longer survival outcomes compared to that of lower RUNX1-RUNX3 expressing cohort, G3: YAP_sig_^high^ Rx1Rx3^low^ (Figure 6B).

Further, single sample GSEA (ssGSEA) analysis [27] was performed on these four cohorts of breast cancer patients to investigate for enrichment of specific gene signatures. YAP_sig_^high^Rx1Rx3^low^ subgroup of patients scored higher enrichment for EMT and stem-ness signatures compared to that YAP_sig_^high^Rx1Rx3^high^ subgroup, while YAP_sig_^low^ subgroup scored the lowest enrichment for both (Figure 6C). EMT and stem-ness gene signature enrichments co-relate with expression levels of YAP-signature in the breast cancer samples.

RUNX1-RUNX3 expression levels show significant effect on survival outcomes of the patients only with high-YAP-signature. And high RUNX1-RUNX3 expression suppresses EMT and stem-ness enrichment specifically in the context of elevated YAP-signature. These findings suggest that RUNX1-RUNX3 expression levels indeed influence prognostic outcomes and metastatic potential of breast cancer patients specifically in the context of elevated oncogenic function of YAP.

YAP1 expression alone did not correlate with grade or survival outcomes of the patients (Supplementary Figure 4C). RUNX1-RUNX3 high or low expression within high YAP1 expression cohort did not correlate with any of the survival outcomes either (Supplementary Figure 4D). This data suggests that not YAP1 expression alone but oncogenic manifestation of YAP function reflected as YAP-signature gene expression is strongly co-related with breast cancer prognostic and metastatic parameters. And together with RUNX1-RUNX3 expression, YAP-signature dictates survival outcomes of breast cancer patients, by modulating EMT and stem-ness gene expression.

## DISCUSSION

Recurrent mutations in RUNX1 [14, 15] and hyper methylation of RUNX3 [26] locus have recently been discovered in breast cancer indicating tumor suppressor role of RUNX1 and RUNX3. However, molecular basis of tumor suppressor function of RUNX1 and RUNX3 in breast cancer is largely unknown. Given that RUNX1 and RUNX3 interact with YAP and co-regulate transcription [11], we investigated whether RUNX1-RUNX3 interaction with YAP has biological significance in the context of breast cancer. Indeed, in mammary epithelial cell lines, co-expression of RUNX1 or RUNX3 significantly suppressed YAP-mediated migration, mammosphere formation and aberrant differentiation of acini in an interaction-dependent manner. Gene expression profiles of mammary epithelial cell lines with stable expression of YAP, with or without RUNX1-RUNX3 clearly demonstrated that co-expression of RUNX1 or RUNX3 antagonized transcriptional profile of YAP-regulated genes in an interaction dependent manner. WW domain of YAP has been implicated in mediating interactions with negative regulators of YAP in MCF10a [28]. Our data identifies RUNX1 and RUNX3 to be one of such negative regulators of YAP in the context of mammary epithelial cells.

Although RUNX1 has been extensively studied as an oncogene as RUNX1-ETO fusion protein in blood cancers, RUNX1's function in breast cancer progression is context dependent. It has been to shown to play an oncogenic role [29], as well as tumor suppressor role in ER^+ve^ breast tumors [30]. While, loss of RUNX1 expression in MCF10a, an ER^−ve^ breast epithelial cell line has been reported to induce EMT via TGFβ and Wnt signaling [31], suggesting that RUNX1 plays a tumor suppressor role in this ER^−ve^ breast epithelial cell line. This report is in concordance with our data where overexpression of RUNX1 in MCF10a suppresses EMT induced by YAP overexpression.

Transcriptional co-operation between YAP and RUNX proteins has been observed earlier. RUNX2, a member of RUNX family that regulates bone morphogenesis has been shown to recruit YAP to the sub-nuclear domains to suppress RUNX2-mediated osteocalcin promoter activation [32]. RUNX1-YAP complex regulates *Itch* transcription, an E3 ubiquitin ligase, which mediates p73 degradation in absence of DNA damage [33]. Studies from our lab in the context of gastric cancer have suggested that RUNX3 delimits DNA binding of TEAD-complex to abrogate pro-tumorigenic activity of YAP-TEAD [18]. Chromatin immunoprecipitation assays for YAP-TEAD-RUNX complex in our stable mammary epithelial cells will provide mechanistic insights, whether co-occupancy of YAP-RUNX at the target promoters is involved in abrogation of YAP-signature gene expression associated with EMT and stem-ness.

Recent reports converge on the finding that YAP is an important player in regulating EMT and stem-ness associated gene expression, and it co-operates with diverse transcription factors like E2F, FOS/AP-1 in a context specific manner. Oncogenic co-operation of YAP with E2F or FOS/AP-1 drives KRAS independent recurrence of KRAS-induced pancreatic and lung tumors [34, 35]. Interestingly, RUNX3 inactivation in KRAS driven lung adenocarcinoma has been shown to accelerate malignant progression [36] and it will be of interest to test, whether RUNX interferes with YAP-FOS/AP1 co-operation in mediating EMT and malignant tumor progression.

In our studies, a comprehensive expression dataset of 3992 breast cancer patients revealed association of elevated YAP-signature with the worse disease outcomes specifically in the context of low RUNX1-RUNX3 expression. Higher expression of RUNX1-RUNX3 proved protective towards shorter survival of high YAP-signature patient cohort. Also, the enrichment pattern for EMT and stem-ness signatures in cell-based model and clinical samples showed stark dependence on high YAP expression, which was abrogated in the context of high RUNX1-RUNX3 expression. Suggesting, that elevated YAP activity may promote EMT and stem-ness in the YAP_sig_^high^ breast tumors. While high RUNX1-RUNX3 expression in YAP_sig_^high^Rx1Rx3^high^ subgroup abrogates EMT and stem-ness function of YAP and leads to longer survival outcomes for YAP_sig_^high^Rx1Rx3^high^ subgroup compared to the rest.

Extension of this study in breast cancer cell lines, revealed 7 of the YAP-Signature targets that are differentially regulated by RUNX3 in high-YAP compared to that in low-YAP context. These include genes already implicated in cancer metastasis and cancer signaling, like Wnt5A [37] CTGF [38], SULF2 [39], integrin B2 [40].

From the study, it is imperative that RUNX3 and RUNX1 regulate YAP-mediated pro-oncogenic phenotypes and YAP-RUNX1-RUNX3 axis has direct implications in breast cancer progression. We anticipate that the functional association between YAP and RUNX proteins can be validated in a larger cohort of breast cancer patients, confirming clinical significance to predict poorer survival outcomes in breast cancer progression. There have been attempts in the recent years to define reliable prognostic tools to predict tumor recurrence using gene signatures. The two successful attempts that FDA approved for deeper expression profiling towards clinical assessment of individual patient are MammaPrint [41] and 21-gene prognostic signature in Oncotype Dx [42]. Successful validation of the clinical significance of YAP-signature and RUNX1-RUNX3 expression can be exploited to define novel prognostic tool to predict survival outcomes of breast cancer patients. Further, studies using RUNX proteins or their peptide derivatives to abrogate YAP-mediated EMT and stem-ness can help find novel ways to target YAP function and thereby enhance survival outcomes of the cancer patients.

## MATERIALS AND METHODS

### Cell culture

MCF10A cells were obtained from ATCC and hTert immortalized HMEC cells were a kind gift from Dr. William Hahn (Dana–Farber Cancer Institute, Boston, MA, USA). Both the mammary epithelial cells were cultured in DMEM/F12 supplemented with 5% horse serum, 20 ng/ml EGF, 0.5 μg/ml hydrocortisone, 100 ng/ml CholeraToxin, 10 μg/ml Bovine Insulin, 100 units/ml penicillin and 100 μg/ml streptomycin [23]. 293T cells were cultured in DMEM supplemented with 10% FBS, 2 mM L-glutamine, 100 units/ml penicillin and 100 μg/ml streptomycin. Cell lines; Hs578T & BT549 were purchased from (ATCC) and were cultured under standard condition in DMEM High Glucose with 10% fetal bovine serum at 37° C and 5% CO_2_.

### Constructs

RUNX3 cDNA (P2 isoform) was cloned in pQCXIP retroviral vector, pmCherry (N3) vector and Doxycycline inducible retroviral vector pRetroX-Tight-pur (Clonetech). RUNX1 cDNA was cloned in pQCXIP. pQCXIH-Myc-YAP (#33091) was obtained from Addgene [43]. pMSCV-YAP S127A; S381A (referred as YAP) and pMSCV-YAP- W199F and W258F (referred as YAP-ww) were generated using PCR-mediated site-directed mutagenesis of the human Flag-YAP construct [6], then sequenced and cloned into MSCV-internal ribosome entry site Hygromycin (MSCV-IRES-Hygro) retroviral vector [6].

### Virus production and stable cell lines

With appropriate GMAC approvals, 293T cells were co-transfected with retroviral construct and packaging vector pCL10A1 using Mirus TransIT-293. Viral supernatants were collected after 48 hrs and filtered through 0.45 μm-disk filter and were stored at **–**80° C. MCF10A and HMEC cells were infected with YAP and/or RUNX3 or RUNX1 retroviruses in 1:1 ration with 8 μg/ml polybrene. Stable cell lines were selected through one passage with 2 μg**/**ml Puromycin and/or 100 μg/ml Hygromycin for MCF10A and 1 μg**/**ml Puromycin and/or 50 μg**/**ml Hygromycin for HMEC. Hs578T and BT549 cell lines were co-infected with pRetroX-Tight-pur-RUNX3 and pRetroX-Tet-on Advance vectors (Clontech). Co-infected cells were selected with 300 μg/ml G418 and 2 μg/ml Puromycin. The cells were induced with 1 μg/ml doxycycline for at least 24 hrs prior to any assay.

### Western analysis of stable cell lines

Whole cell lysates of stable cell lines were extracted from cell pellets with equal volumes of modified RIPA buffer (20 mM Tris-HCl pH 8.0, 420 mM NaCl, 10% Glycerol, 0.5% NP-40, 0.1 mM EDTA, with 1mM DTT, 10 mM PMSF, 20 mM protease inhibitor). Extracts were then centrifuged at 13,500 *g* for 15 minutes and supernatants were analyzed by immunoblotting. The list of antibodies used is provided in Table 1.

**Table 1:** List of antibodies used

1° Antibody	Dilution	2° Antibody	Dilution	Source
RFP	1:1000	anti-mouse HRP	1:10,000	MBL
FLAG	1:1000	Anti-rabbit HRP	1:10,000	Sigma-Aldrich
Actin	1:1000	anti-mouse HRP	1:10,000	Sigma-Aldrich
a-tubulin	1:1000	anti-mouse HRP	1:10,000	Sigma-Aldrich
E-Cadherin	1:1000	anti-mouse HRP	1:10,000	BD Bioscience
Fibronectin	1:1000	anti-rabbit	1:10,000	Santa Cruz
GAPDH	1:1000	anti-mouse HRP	1:10,000	Santa Cruz
N-Cadherin	1:1000	anti-mouse HRP	1:10,000	Santa Cruz
5G4-RUNX3	1:1000	anti-mouse HRP	1:10,000	Y. Ito Lab
RUNX3	1:1000	anti-rabbit	1:10,000	Cell-Signaling
YAP	1:1000	anti-rabbit	1:10,000	Cell-Signaling

### Cell viability

Stable MCF10a cells were plated onto 96-well plate in triplicate wells with density 10^3^ cells per well. Cell viability was measured in complete growth medium using WST reagent at 450 nm on day 1, 2 and 3. Day 3 readings from three independent experiments were normalized to vector control cell line and their averages with standard error of mean are plotted using graph pad prism.

### Trans-well migration assay

Sub-confluent cultures of MCF10a or HMEC stable cell lines were starved in EGF free assay media overnight. 50K cells were plated in triplicates on the 8 μm pore size trans-well chambers (BD Biosciences) in EGF free assay media and were allowed to migrate towards complete media. 24 h later, the cells were fixed with 1% PFA and permeabilized with 0.1% tween-20. The chambers were then cotton swabbed and stained with DAPI. Cut inserts were mounted on the slides and were imaged and analyzed using Zeiss Axiovision software.

Hs578T and BT549 stable cell lines were cultured with or without 1 μg/ml doxycycline for 24 hrs. The cells were then serum starved overnight. 50 K cells in serum free media were allowed to migrate to complete media. The inserts were fixed and migrated nuclei were counted as above.

### Co-Immunoprecipitation

293T cells were transfected with respective constructs using Mirus TransIT-293. 48 hr after transfection, nuclear extracts were harvested using NE-PER Nuclear and Cytoplasmic Extraction Reagents (Pierce), and were dialyzed for 1 hour in Dialysis Buffer (20 mM HEPES pH 7.5, 100 mM KCl, 1 mM EDTA, 1 mM EGTA). 1–2 mg/ml nuclear extracts were nutated for 2 hrs with 25 μl of Flag-beads or RFP-trap beads (Chromotek) following recommended protocol. The beads were washed with 250 mM NaCl buffer five times (20 mM HEPES pH 7.9, 250 mM NaCl, 10% glycerol, 0.2% NP-40, 0.1 mM EDTA) followed by one wash with 150 mM NaCl buffer (20 mM HEPES pH 7.9, 150 mM NaCl, 10% glycerol, 0.1% NP-40, 0.1 mM EDTA). For Flag-IP, co-immunoprecipitated proteins were eluted with Flag-peptide competition followed by boiling the beads with Laemmli sample buffer. Elutes were then resolved on 10% SDS-PAGE gel for immunoblotting. The antibodies used are listed in Table 1.

### Mammosphere assay

For MCF10a stable cells, single cells were plated in ultralow attachment 6-well plates (Corning) in triplicates at a density of 10,000 cells/ml and cultured with 1.5% Methocel (Sigma) in DMEM: F12 medium (serum free) supplemented with B27 (Invitrogen) in 1:50 dilution, 20 ng/ml EGF and bFGF and 1 μg/ml hydrocortisone, 5 μg/ml bovine insulin. Every 3 days, 500 μl fresh media was added. On day 11, all the Mammosphere were counted and their diameter was measured using an ocular at 4X magnification. DIC images were taken at 4X magnification on day 11. Average number of mammosphere; greater than 50 μm size from two independent experiments are plotted using graph pad prism.

Mammosphere from each stable cell line were collected on day 11 and total protein was extracted using modified RIPA buffer. Western analysis was performed for YAP, RUNX1 and RUNX3 and actin expression.

For Hs578T and BT549 cell lines, the cells were grown in +/− 1 μg/ml doxycycline for 24 hrs and then plated as single cells onto ultralow attachment 96 well plate (Corning) in triplicates at a density of 1000–2000 cells in the same media as above. Numbers of mammosphere that are larger than 50 μm size were counted on Day 11 from three independent experiments.

### 3D mammary acini culture on matrigel for morphogenesis assay

8-well chamber slides (BD Biosciences) were coated with 30μl of growth factor reduced matrigel (BD Biosciences). 5000 cells were plated per well in assay media containing 2% growth factor reduced matrigel (BD Biosciences) and 5 ng/ml EGF. The medium was replaced every 3 days. On 12th day images were taken and using Olympus Cell^F^ software diameter was measured and recorded for 100–150 acini for each stable cell line. Statistical analysis was performed with Prism software.

### Confocal microscopy of 3D mammary acini

On day 16th, mammary acini were fixed in 2% PFA and were permeabilized with 0.5% Triton-X. Permeabilized acini were then stained with 1:100 Rhodamine-phalloidin (Molecular Probes) for 1 hr. Acini mounted in Prolong gold, with DAPI (Life technologies) mounting media were then imaged with Nikon A1R confocal microscope.

### Microarray expression analysis

RNA was isolated from sub-confluent cultures of MCF10A stable cell lines using RNeasy Mini kit (Qiagen) followed by on column DNAse I treatment. The quality of RNA was assessed using Bioanalyzer (Agilent Technologies, Santa Clara, CA, USA) with the RNA 6000 Nano kit. Only RNA with RNA integrity number value greater than 8 and with a 28S rRNA band at 4.9 kb that is twice that of the 18S rRNA band at 1.9 kb, was selected for analysis. Applause WT-Amp ST system (NuGEN, San Carlos, CA, USA) was used to produce amplified cDNA from 200 ng of total RNA following the manufacturer's protocol. Encore Biotin Module (NuGEN) was used to performed cDNA fragmentation and biotin labeling using 2 μg of amplified cDNA. Biotin labeled cDNA was then mixed with hybridization cocktail which contained 1.8 μL of control oligonucleotide B2 (3 nM), 5.5 μL of 20× Eukaryotic hybridization controls, 55 μL of hybridization buffer and 11 μL of DMSO (all from Affymetrix, Santa Clara, CA, USA). The prepared targets were hybridized overnight to Affymetrix Human Gene 1.0ST array. Following hybridization, Gene chips were washed, stained and scanned according to the protocol described in WT Sense Target Labeling Assay Manual (FS450_0007). CEL files were normalized using RMA algorithm. All of the microarray raw data tables have been deposited in the Gene Expression Omnibus under the accession number GSE60876.

### Fluidigm RT-PCR

RNA was extracted from cells using RNeasy Mini Kit (Qiagen) according to the manufacturer's instructions. Reverse transcription was performed using the fluidigm reverse transcription master mix using a RNA concentration of 200 ng/ul. The prepared cDNA was subjected to pre-amplification using the PreAmp Master Mix (Fluidigm PN 100–5580) with a pooled DELTAgene Assay Mix (500 nM), which was prepared using 1ul of each 100 μM stock primer. Prior to RT-PCR, pre-amplified cDNA were treated with exonuclease I treatment (New England BioLabs, PN M0293L) to remove unincorporated primers with the final products diluted by 5 fold using TE Buffer (10 mM Tris-HCl; 1.0 mM EDTA). A 48.48. Dynamic Array™ IFC was used with 2x SsoFast EvaGreen Supermix with Low ROX (Bio-Ras, PN 172-5211) along with 20X DNA Binding Sye Sample Loading Reagent (Fluidigm, PN-100 3738). The results obtained were analyzed using Fluidigm Real Time-PCR analysis.

Normalization of Fluidigm RT-PCR was computed using CT values with respect to housekeeping genes: *B2M*, *HMBS*, *PGK1*, *SDHA*, *TBP*, and *YWHAZ*. These housekeeping genes were confirmed to have coefficient of variation less than 0.1 across samples. Post housekeeping normalization, the ΔΔCT was computed with respect to non-template control.

### Analysis of breast cancer patients’ Affymetrix microarray gene expression

26 breast cancer cohorts on Affymetrix U133A or U133Plus2 were downloaded from Gene Expression Omnibus (GEO) and ArrayExpress. This panel of 26 cohorts comprises 3992 human breast tumor samples, including E-TABM-158 (*n* = 130), GSE11121 (*n* = 200), GSE12276 (*n* = 204), GSE1456 (*n* = 159), GSE1561 (*n* = 49), GSE19615 (*n* = 115), GSE20181 (*n* = 176), GSE2034 (*n* = 286), GSE21653 (*n* = 266), GSE23177 (*n* = 116), GSE23593 (*n* = 50), GSE23988 (*n* = 61), GSE25066 (*n* = 508), GSE26639 (*n* = 226), GSE31519 (*n* = 67), GSE3494 (*n* = 251), GSE3744 (*n* = 47), GSE4922 (*n* = 40), GSE5327 (*n* = 58), GSE5460 (*n* = 127), GSE5764 (*n* = 10), GSE6532 (*n* = 414), GSE6596 (*n* = 24), GSE7390 (*n* = 198), GSE9195 (*n* = 77), and HESS cohort (*n* = 133) [44]. Out of the 3992 tumor samples, 974 have overall survival information, and 2,333 have disease-free survival information. Robust Multichip Average (RMA) normalization was performed on each cohort and subsequently, the normalized data was standardized using ComBat [45] to remove batch effect.

### Statistics

Statistical significance evaluation was computed using Matlab^®^ R2012a and Graphpad Prism^®^ version 5.0. Significance was calculated using Students’ *t*-test or one way Anova for multiple comparisons followed by Newman–Keuls multiple comparison test, where ^*^p < 0.05, ^**^p < 0.005 and ^***^p < 0.0005. Kaplan–Meier analyses were performed using Graphpad Prism^®^ version 5.0 and significance was determined using log-rank test.

### Study approval

GMAC: For generation of retroviral constructs expression YAP, RUNX1 and RUNX3 proteins in mammary epithelial cell lines, approval was obtained from “Genetic Modification Advisory Committee” of Singapore.

## SUPPLEMENTARY MATERIALS FIGURES AND TABLES








