# Supplementary material for: RUNX1 and RUNX3 protect against YAP-mediated EMT, stem-ness and shorter survival outcomes in breast cancer
Source: Oncotarget. 2018 Feb 6;9(18):14175–92. doi: 10.18632/oncotarget.24419 (PMC5865662; doi:10.18632/oncotarget.24419)
Supplement: Supplementary file 1 [file oncotarget-09-14175-s001.pdf]

RUNX1 and RUNX3 protect against YAP-mediated EMT, stem-ness and shorter survival outcomes in breast cancer

SUPPLEMENTARY MATERIALS

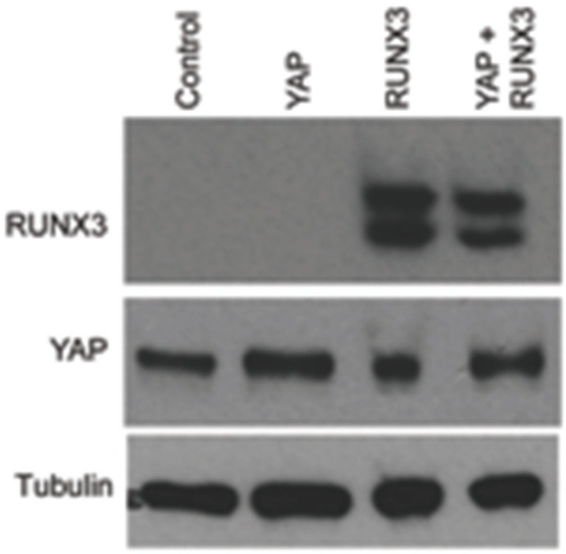

Supplementary Figure 1: YAP and RUNX3 expression analyzed for MCF10a stable cell lines. Western analysis of 20 μg whole cell extracts from stable MCF10a cell lines. The lanes from left to right: MCF10a stably infected with vector control, YAP, RUNX3 and YAP+RUNX3. Western analysis was performed with YAP and RUNX3 antibody with tubulin as a loading control.

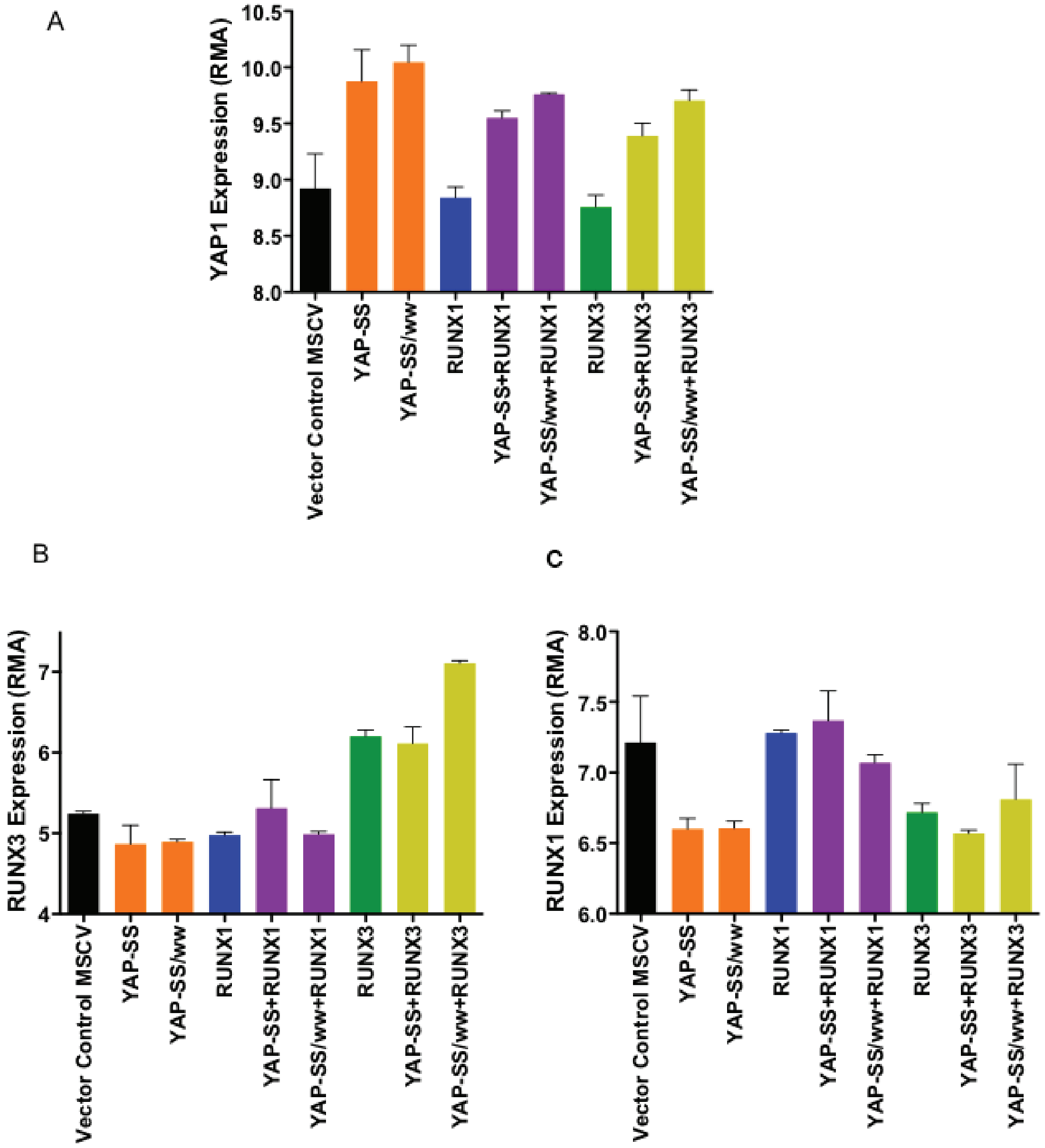

Supplementary Figure 2: Microarray expression of YAP, RUNX1 and RUNX3 for MCF10a stable cell lines. Microarray analysis was performed using Affymetrix Human Gene 1.0 ST array for MCF10a stable cell lines expressing YAP and/or RUNX1 and RUNX3 (See supplementary methods). After RMA normalization expression of (**A**) YAP, (**B**) RUNX3 and (**C**) RUNX1 was assessed in each stable cell line.

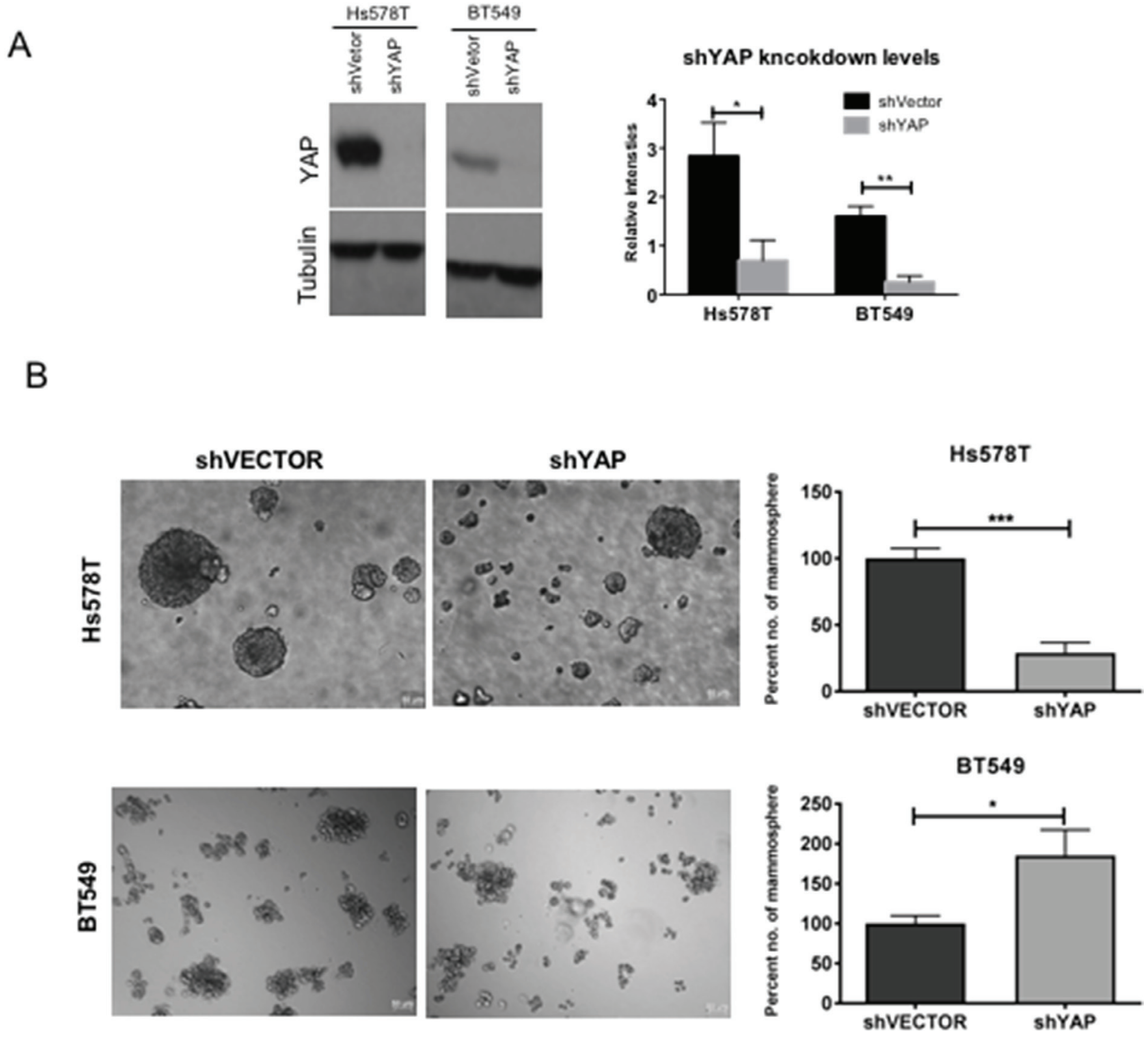

Supplementary Figure 3: YAP knock-down in cancer cell lines with differential YAP expression Hs578T and BT549 cell lines were infected with lentivirus expressing either shVector or shYAP. Stable cell lines were analyzed for effect of YAP knock-down. The error bars in the plots represent standard error of mean. Statistical significance is calculated using unpaired student's *t*-test, where *is $p < 0.05$, ** is $p < 0.005$ and *** is $p < 0.005$. (**A**) 30μg of whole cell lysates is run for western analysis of stable cell lines to assess YAP protein expression compared to that of tubulin as a loading control. Average expression of YAP in shYAP stable cell lines normalized to shVector stable cell lines are plotted from three independent western blots. (**B**) Average number of Mammosphere (>50 μm) counted on day 11, from triplicate experiments are plotted for shVector and shYAP stable cell lines. The numbers are normalized to mean mammosphere formed by shVector stable cell line.

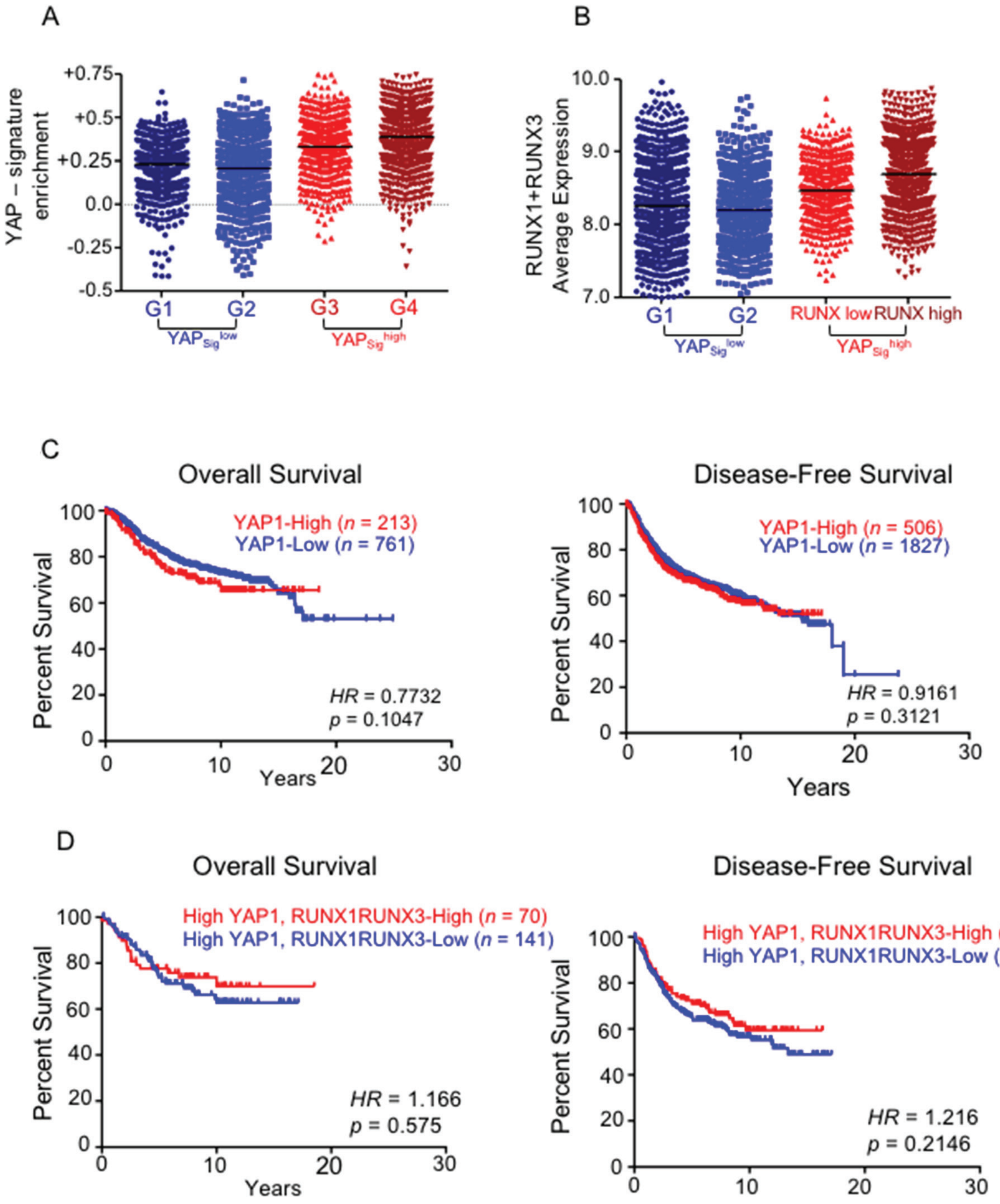

Supplementary Figure 4: Survival curves of 3992 breast cancer patients stratified by YAP expression and YAP-Signature gene expression. Microarray expression data of 3992 breast cancer patients was compiled and normalized. YAP, RUNX1-RUNX3 average expression levels in the tumors were compared to that of normal mammary tissue and the patient samples were segregated into two cohorts with higher (High) or lower (Low) than normal expression. Survival analysis was carried out to assess association and hazard ratios and significance are indicated. (**A**) Individual patient sample from the cohort of 3992 breast cancer patients was subjected to unsupervised hierarchical clustering based on the expression of YAP-signature genes. The four clusters that were generated by Cluster 3.0 software were assessed for the enrichment score of the YAP-signature by ssGSEA. The distribution of the enrichment scores within the four groups (G1 to G4) is plotted. Based on the median expression, the group G1 and G2 are referred as YAP-Signature^{low} and group G3 and G4 are referred as YAP-Signature^{high}. (**B**) Average gene expression of RUNX1 and RUNX3 in individual patient sample is plotted for each of the four subgroups as categorized in Supplementary Figure 4C. Amongst the YAP-Signature^{high} group (G3 and G4), G4 is referred as YAP-Signature^{high} and Rx1Rx3^{high} (color code; maroon) and G3 is referred as YAP-Signature^{high} and Rx1Rx3^{low} (color code; red) based on the median RUNX1-RUNX3 average expression. (**C**) Kaplan-Meier analysis for patients with High and Low YAP expression towards overall and disease-free survival. (**D**) Kaplan-Meier analysis to assess the association of RUNX1-RUNX3 expression levels within high-YAP and low-YAP cohorts with overall and disease-free survival.

Supplementary Table 1: YAP-Signature. See Supplementary_Table_1

Supplementary Table 2: Of significance test for figure 4A. See Supplementary_Table_2

Supplementary Table 3: Gene list in 5E and statistics for 5F. See Supplementary_Table_3
